# Effect of an Oral Adsorbent, AST-120, on Dialysis Initiation and Survival in Patients with Chronic Kidney Disease

**DOI:** 10.1155/2012/376128

**Published:** 2012-01-12

**Authors:** Shingo Hatakeyama, Hayato Yamamoto, Akiko Okamoto, Kengo Imanishi, Noriko Tokui, Teppei Okamoto, Yuichiro Suzuki, Naoaki Sugiyama, Atsushi Imai, Shigemasa Kudo, Takahiro Yoneyama, Yasuhiro Hashimoto, Takuya Koie, Noritaka Kaminura, Hisao Saitoh, Tomihisa Funyu, Chikara Ohyama

**Affiliations:** ^1^Department of Advanced Transplant and Regenerative Medicine, Hirosaki University Graduate School of Medicine, 5 Zaifu-chou, Hirosaki 036-8562, Japan; ^2^Department of Urology, Hirosaki University Graduate School of Medicine, Hirosaki 036-8562, Japan; ^3^Department of Urology, Oyokyo Kidney Research Institute, Hirosaki 036-8243, Japan

## Abstract

The oral adsorbent AST-120 has the potential to delay dialysis initiation and improve survival of patients on dialysis. We evaluated the effect of AST-120 on dialysis initiation and its potential to improve survival in patients with chronic kidney disease. The present retrospective pair-matched study included 560 patients, grouped according to whether or not they received AST-120 before dialysis (AST-120 and non-AST-120 groups). The cumulative dialysis initiation free rate and survival rate were compared by the Kaplan-Meier method. Multivariate analysis was used to determine the impact of AST-120 on dialysis initiation. Our results showed significant differences in the 12- and 24-month dialysis initiation free rate (*P* < 0.001), although no significant difference was observed in the survival rate between the two groups. In conclusion, AST-120 delays dialysis initiation in chronic kidney disease (CKD) patients but has no effect on survival. AST-120 is an effective therapy for delaying the progression of CKD.

## 1. Introduction

The number of dialysis patients is increasing worldwide [1] as a consequence of treatment to prevent progression of chronic kidney disease (CKD) remaining largely unresolved. To prevent CKD progression from an early stage, many clinical studies have suggested that disease progression may be curbed by controlling dietary factors, blood pressure, lipids, anemia, and mineral balance [2]. However, management of protein and salt restriction along with blood pressure is not always effective in patients using a multidisciplinary approach, including the use of angiotensin-converting enzyme inhibitors (ACEIs) and angiotensin receptor blockers (ARBs), that forms the basic strategy currently used to control CKD progression [1, 3]. This suggests the necessity for further development of modalities to control CKD progression.

 AST-120 (Kremezin, Kureha Corporation, Tokyo, Japan) is an oral adsorbent consisting of microspheres made from porous carbon material [4]. By adsorbing uremic toxins, including indoxyl sulfate, AST-120 can slow CKD progression and delay dialysis initiation. The drug has been clinically used in Japan since 1991 and is covered by health insurance [5, 6].

Although large-scale multicenter trials have shown the effect of AST-120 to delay dialysis initiation in CDK patients, these reports were published in the late 1980s [7]. Updating data on the AST-120 efficacy for delaying dialysis initiation is therefore necessary under the currently employed basic strategy. Recently, Ueda et al. [8] and Maeda et al. [9] reported the efficacy of AST-120 on CKD progression and delay in dialysis initiation. These studies used the propensity score matching method to compare the efficacy of AST-120, although the number of patients studied was relatively small.

 The importance of early diagnosis and treatment of CKD patients is well established as cardiovascular disease is known to be the primary cause of high mortality in these patients. Recently, Ueda et al. [10] reported improved prognosis in CKD patients who had been administered AST-120 during the predialysis period. However, further evidence is required to elucidate the effect of AST-120 on the prognosis of dialysis patients [11].

 In the present study, we performed a retrospective examination on the therapeutic effect of AST-120 in combination with current basic treatment regimens in CKD patients. To guarantee the validity of the retrospective analysis we used the propensity score method to identify a matched-control group.

## 2. Patients and Methods

Between January 1991 and December 2010, 872 of CKD patients were followed until dialysis initiation at Oyokyo Kidney Research Institute, Hirosaki. Of the 872 patients, 363 had a history of using AST-120, and 509 patients had never received AST-120. By applying the propensity score matching method, 560 patients were pair-matched (*n* = 280 of each group) and enrolled in the study. Dialysis initiation was determined based on scores consisting of (1) clinical symptom (fluid retention, electrolyte abnormality, gastrointestinal symptoms, circulatory symptoms, neurological symptoms, hematological disorders, and vision disorders), (2) remnant kidney function (serum creatinine or creatinine clearance), (3) impairment level of daily living, (4) age (10 years or less, and 65 or older), and (5) existence of systemic vascular disorder according to the guidelines for introducing patients to dialysis issued by the Ministry of Health, Labour and Welfare of Japan. Patients with sum total 60 or higher were indication for dialysis initiation (Table 1) [12]. This study was approved by the institutional ethical committee of Oyokyo Kidney Research Institute.

### 2.1. Pair-Matching Methods

To guarantee the validity of this retrospective analysis, a propensity score was applied to pair-matched patients. Propensity scores were calculated using logistic analysis. The data used in the analyses included age, gender, blood pressure, biochemistry, concomitant drugs (activated vitamin-D, ACEIs, ARBs, and calcium blockers), estimated glomerular filtration rate (eGFR), presence of diabetes mellitus, and cardiovascular disease (heart failure, myocardial infarction and angina pectoris) at the initial visit. The eGFR was calculated using age, gender, and serum creatinine (sCr) by the equation shown below [13]. This eGFR equation for Japanese patients is a modified version of the abbreviated Modification of Diet in Renal Disease Study formula [14]. [eGFR mL/min/1.73 m^2^ = 194 × sCr^−1.094^ × age^−0.287^ (×0.739, if female)]. AST-120 administration quitted after the initiation of hemodialysis.

 Based on the scores of each patient, two patients with a score within 0.03 were selected as a pair. For the AST-120 group, the baseline was defined as the time of initiation of AST-120 treatment. Baseline for patients in the control group was defined as the date of measurement of eGFR that was closest to the baseline eGFR level of their counterpart in the AST-120 group.

 We compared the effect of AST-120 to delay dialysis initiation in patients taking AST-120 (AST-120 group) with pair-matched patients not taking AST-120 (non-AST-120 group). Because all patients received dialysis, the 12- and 24-month dialysis initiation free rate was assessed as primary endpoints between the pair-matched AST-120-treated and non-AST-120 treated groups. The 3-, 5-, and 10-year survival rate after administration of AST-120 was assessed as a secondary endpoint.

### 2.2. Evaluation

The background clinical data and concomitant drugs of the two groups were compared using the chi-square test. Age and other biochemical parameters were expressed as mean ± SD, and statistical differences were calculated by Student's *t*-test. The level of urinary protein excretion was expressed as median and tested by Mann-Whitney's *U*-test. Cumulative dialysis initiation free rate and survival rate were plotted by the Kaplan-Meier method, and intergroup differences were tested by the log-rank test. A *P* value of less than 0.05 was considered significant. Cox regression adjusted for these factors was also performed. All analyses were performed using SPSS (SPSS Inc, ver. 12.0, Chicago, IL, USA).

## 3. Results

The baseline characteristics of the matched patients are summarized in Table 2. After matching, no significant differences were observed in patients' background between the two groups. At the time of dialysis initiation, the effect of AST-120 on blood pressure, blood, and serum data were compared between AST-120 and non-AST-120 group (Table 3). There was no difference in blood and serum data but systolic and diastolic blood pressure showed significant difference between two groups (*P* = 0.0217 and *P* = 0.0180, resp.)

**Figure 1:**
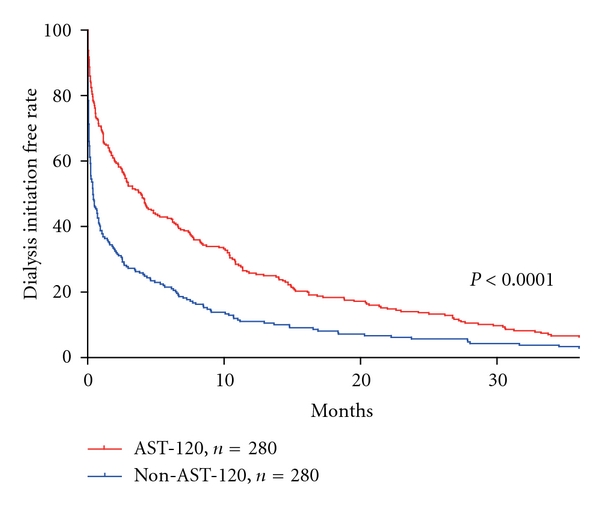
The delaying effect of AST-120 on dialysis initiation. The cumulative percentage of nondialysis patients analyzed by Kaplan-Meier methods showed the delaying effect of AST-120. The dialysis initiation free rate was significantly higher in the pair matched patients receiving AST-120 (AST-120 group, *n* = 280) compared to those not receiving AST-120 (non-AST-120 group, *n* = 280) (*P* < 0.001).

The effect of AST-120 on dialysis initiation in the pair-matched patients is shown in Figure 1. The 12- and 24-month dialysis initiation free rate was significantly higher in the AST-120 group (25.0% and 13.7%, resp.) than in the non-AST-120 group (10.5% and 5.7%, resp.) (Figure 1, Table 4).

**Figure 2:**
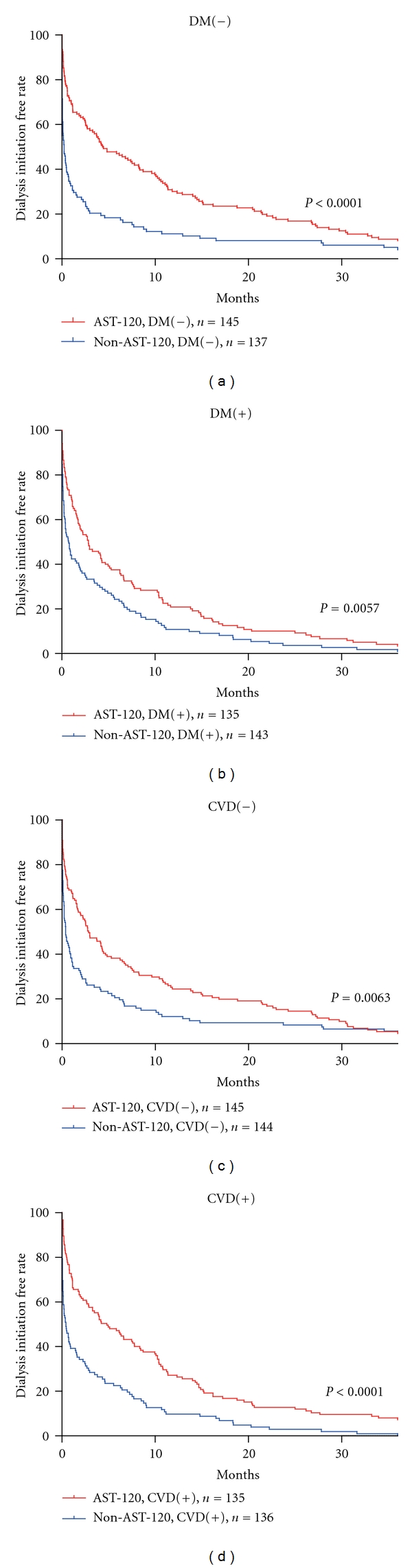
Subgroup analysis of the delaying effect of AST-120 on dialysis initiation. The cumulative percentages of non-dialysis patients were analyzed by Kaplan-Meier methods. The dialysis initiation free rate was significantly higher in the AST-120 group than in the non-AST-120 group in all subgroups, with DM [11] (a) or without DM (b), and with CVD (c) or without CVD (d).

Using subgroup analysis, we evaluated the efficacy of AST-120 in patients with diabetic nephropathy or nondiabetic renal disease, and also with or without cardiovascular disease. For patients with diabetic nephropathy or cardiovascular disease, the 12- and 24-month dialysis initiation free rate was significantly higher in the AST-120 group than in the non-AST-120 group (Figure 2, Table 4). This delay in dialysis initiation was longer in patients with cardiovascular disease or those without diabetic nephropathy.

We performed a Cox's proportional hazard model based on uni- and multivariate analysis to determine the independent factors for dialysis initiation in the pair-matched patients. For univariate analysis we selected eGFR and not receiving AST-120 as independent factors for increasing the risk for dialysis initiation. Similarly, for multivariate analysis we selected anemic status, eGFR, and not receiving AST-120 as independent factors associated with a significant increase in risk of dialysis initiation (Table 5).

**Figure 3:**
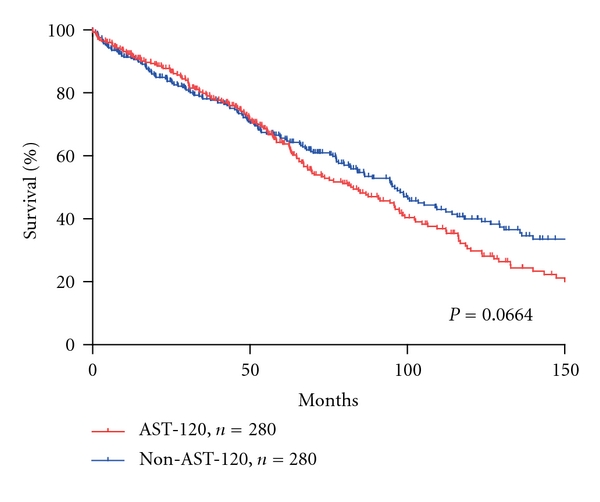
Patient survival rate after the administration of AST-120. Patient survival rate was not significantly different in the pair-matched patients with or without AST-120 treatment.

 To examine whether or not the use of AST-120 in the predialysis period influenced prognosis after dialysis initiation, we compared the 3-, 5-, and 10-year survival rate from administration of AST-120 by the Kaplan-Meier method with a log-rank test. We found that there was no significant difference in survival rates between the AST-120 and non-AST-120 groups (*P* = 0.0664) (Table 6, Figure 3).

## 4. Discussion

CKD is recognized as a disease that increases the risk of many adverse events closely associated with death, such as cardiovascular disease [15]. Early diagnosis and treatment are necessary to delay CKD and prevent these adverse events. AST-120 is an oral adsorbent that slows the progression of CKD by decreasing serum nephrotoxic substances such as indoxyl sulfate. Recently, the CAP-KD study was conducted to evaluate the usefulness of AST-120 in patients with moderate to severe CKD [16]. This study showed no significant difference in the composite primary endpoints (doubling of sCr level, increase in sCr level ≥ 6.0 mg/dL, need for dialysis or transplantation, and death), but revealed a significant suppression in the decrease of estimated GFR over 56 weeks in the AST-120 group. However, because of the short follow-up period, the CAP-KD study could not clarify the impact of AST-120 on the development of end-stage renal disease [16].

 In the present study, we retrospectively analyzed the effects of AST-120 to delay dialysis initiation and survival in the context of current treatment regimens. Our results showed a significantly lower rate of dialysis initiation in the AST-120 group. This indicated that AST-120 is a useful treatment and supports recent retrospective studies by Ueda et al. [8] and Maeda et al. [9] that also showed AST-120 delayed dialysis initiation. Furthermore, Maeda et al. [17] reported long-term treatment of chronic renal failure with AST-120 from early CKD stage has potential to slowing progression of renal failure and delaying initiation of dialysis. In this present study, our result has limitation because main patients were at the late stage of CKD (CKD stage 5) and half of patients are initiated to hemodialysis within half year. These results suggest that AST-120 administration from early CKD stage has potential to improve the progression of renal failure effectively.

The present study had some limitations. Although the pair-matching method minimizes imbalances in patient background, the finding that AST-120 delayed dialysis initiation is not definitive, given that this was a retrospective study on a relatively small number of patients from a single institute. It is possible that the better outcomes in the AST-120 group may be due to selection bias, and therefore a prospective, randomized trial with long-term followup is essential to evaluate the effectiveness of AST-120. In addition, we could not evaluate the effects of AST-120 on blood and serum data from AST-120 administration to dialysis initiation. Our data showed significant decrease in blood pressures at the time of dialysis initiation, but we could not conclude the effect of AST-120 on blood pressures because of retrospective limitations. Recently, Nakamura et al. [18] published AST-120 effects on sCr, eGFR, serum interleukin-6 (IL-6), proteinuria, and urinary excretion levels of 8-hydoxydeoxyguanosine (8-OHdG) and L-fatty acid binding protein (L-FABP), markers of oxidative stress and tubular injury. In their prospective study, 50 patients were divided in to 2 groups (AST-120 and non-AST-120 group, *n* = 25 each) and followed up for 12 months. They showed AST-120 treatment significantly reduced sCr, eGFR, IL-6, proteinuria 8-OHdG, and L-FABP, but no effect on blood pressure. This study suggests AST-120 has potential to protect kidney from stresses and injury.

 In addition to its known effect of delaying dialysis initiation, the influence of AST-120 on subsequent prognosis requires further study. Recent studies suggested that high levels of serum indoxyl sulfate, one of the substances known to enhance progression of atherosclerotic lesions, increase the risk of a cardiovascular event or death in dialysis patients [19]. These studies showed an association between the serum levels of indoxyl sulfate and mortality in CKD patients, indicating that this nephrotoxic compound may be involved in CKD progression and vascular disease [19]. Ueda et al. [10] suggested that patients receiving AST-120 before dialysis initiation tend to have an improved prognosis. In their report, the 5-year survival rate was significantly higher in the AST-120 group (72.6% in the AST-120 group and 52.6% in the non-AST-120 group). However, our results showed no significant difference in survival rates between the pair-matched AST-120 and non-AST-120 groups. This conflict result might be caused by the initial CKD stage difference in AST-120 administration. In the present study, 94% of patients were stage 5 (eGFR, 8.0 ± 5.7 mL/min) but CKD stage of study population in Ueda et al. [10] was stage 4 (average of eGFR, 24-25 mL/min). Yu et al. [20] prospectively investigated the role of indoxyl sulfate in endothelial dysfunction in CKD stage 4 patients and reported that AST-120 administration improved endothelial dysfunction associated with a decrease in indoxyl sulfate and a restoration of antioxidant reserve. H. Shibahara and N. Shibahara [21] reported AST-120 administration contributed to the improvement of cardiac and renal function in moderate CKD patients (sCr, 1.3–2.0 mg/dL) in their prospective study. In addition, Nakamura et al. [18] evaluated AST-120 effects for tubular damage through the reduction of proteinuria and oxidative stress generation in CKD stage 4 patients. From these observations, we speculate that long-term AST-120 administration from early CKD stage might be essential to improve patient survival after dialysis initiation. However, this contradictory result suggests that multiple factors may have a considerable impact on patient survival and also that there may be difficulties with retrospective analysis of CKD patients with heterogeneous backgrounds. Prospective trials are therefore necessary to confirm the beneficial effects of AST-120 on patient survival after dialysis initiation.

## 5. Conclusions

Although the present study is retrospective and has some limitations, our results support the evidence that AST-120 treatment is associated with significant delays in the cumulative dialysis initiation rate. However, it has no effect on patient survival after dialysis initiation. A prospective randomized study may be necessary to probe survival benefit.

## Figures and Tables

**Table 1:** Dialysis initiation criteria in Japan. According to the guidelines for introducing patients to dialysis issued by the Ministry of Health, Labour and Welfare of Japan, sum total of 60 or higher is indication for dialysis initiation.

Items		Score
	Fluid retention	
	Electrolyte abnormality	
	Gastrointestinal symptoms	
	Circulatory symptoms	
Clinical symptoms	Neurological symptoms	
	Hematological disorders	
	Vision disorders	
	High grade	30
	Intermediate grade	20
	Low grade	10

	serum creatinine (mg/d)	
	(creatinine clearance : CCr (mL/min))	
Remnant Renal function	sCr 8 mg/mL or higher (CCr <10 mL/min)	30
	sCr 5*∼*8 mg/mL (CCr 10*∼*20 mL/min)	20
	sCr 3*∼*5 mg/mL (CCr 20*∼*30 mL/min)	10

	High grade: impossible to wake-up	30
Impairment level of daily living	Intermediate grade: marked limitation of activity	20
	Low grade: slight limitation of activity	10

Age	10 years or less	10
	65 years or older	10

Systemic vascular disorder	Existence of systemic vascular disorder	10

**Table 2:** Characteristics of 560 pair-matched patients. No significant differences were observed in the backgrounds of the two groups. CKD: chronic kidney disease, BMI: body mass index, Hb: hemoglobin, eGFR: estimated glomerular filtration rate, Alb: albumin, IP: inorganic phosphate, Ca: calcium, IP: inorganic phosphorus, BP: blood pressure, CVD: cardiovascular disease, DM: diabetes mellitus, Vit D: vitamin D, ACEIs: angiotensin-converting enzyme inhibitors, ARBs: angiotensin receptor blockers.

	All	non-AST-120	AST-120	*P* value
*n*	560	280	280	
Age	66.2 ± 12.3	66.4 ± 12.0	66.1 ± 12.6	0.7869
Gender (M/F)	345/215	181/99	164/116	0.1396
CKD stage, *n* (%)				
3	4 (0.7)	3 (1.1)	1 (0.4)	
4	19 (3.4)	14 (5.0)	15 (5.3)	0.5956
5	527 (94.0)	263 (93.9)	264 (94.3)	
Baseline data				
BMI	23.7 ± 4.1	23.7 ± 4.2	23.7 ± 4.0	0.8642
Hb (g/dL)	8.8 ± 1.7	8.9 ± 1.8	8.8 ± 1.6	0.6265
eGFR (mL/min)	8.0 ± 5.7	8.0 ± 6.9	8.0 ± 4.2	0.8847
Alb (g/dL)	3.6 ± 0.6	3.6 ± 0.6	3.6 ± 0.6	0.9107
IP (mg/dL)	5.4 ± 1.5	5.4 ± 1.6	5.5 ± 1.5	0.8662
Corrected Ca (mg/dL)	8.4 ± 1.1	8.4 ± 1.0	8.4 ± 1.1	0.9841
Systolic BP (mmHg)	160 ± 27.3	160 ± 27.7	159 ± 27.0	0.706
CVD (−/+), *n*	289/271	144/136	145/135	0.9326
DM (−/+), *n*	282/278	137/143	145/135	0.4989
Use of activated Vit D (−/+), *n*	172/388	88/192	84/196	0.714
Use of ACEls/ARBs (−/+), *n*	182/378	92/188	90/190	0.8568
Use of Ca Blocker (−/+), *n*	73/478	36/244	37/243	0.9001
Urine protein (mg/dL) (median)	264	200	270	0.2842

**Table 3:** The effect of AST-120 on blood pressure, blood, and serum data at the time of dialysis initiation.

At the time of HD initiation	All	non AST-120	AST-120	*P* value
Hb (g/L)	8.6 ± 1.5	8.5 ± 1.6	8.7 ± 1.5	0.1276
BUN (mg/dL)	96.6 ± 29.9	94.4 ± 28.6	98.6 ± 31.1	0.1786
Serum creatinine (mg/dL)	8.7 ± 4.8	8.4 ± 2.8	9.0 ± 6.1	0.1702
IP (mg/dL)	6.2 ± 1.6	6.1 ± 1.6	6.3 ± 1.6	0.4590
Total protein (g/dL)	6.1 ± 0.8	6.1 ± 0.9	6.1 ± 0.8	0.6722
Albumin (g/dL)	3.4 ± 0.6	3.4 ± 0.6	3.4 ± 0.6	0.5151
Corrected Ca (mg/dL)	8.5 ± 1.1	8.5 ± 1.1	8.6 ± 1.1	0.6528
Systolic BP (mmHg)	162 ± 25.5	165 ± 26.5	159 ± 24.2	0.0217
Diastolic BP (mmHg)	83.2 ± 13.8	85.0 ± 14.1	81.5 ± 13.3	0.0180

**Table 4:** The 12- and 24-month dialysis initiation free rate. The dialysis initiation free rate was significantly higher in the AST-120 group in all the pair-matched patients and also subgroups with or without DM or CVD.

Dialysis initiation free rate (%)	12 months	24 months	*P* value
AST-120	25.0	13.7	<0.0001
non-AST-120	10.5	5.7	
AST-120, DM(−)	30.1	17.6	<0.0001
non-AST-120, DM(−)	11.2	8.2	
AST-120, DM(+)	20.8	10.0	0.0057
non-AST-120, DM(+)	10.8	3.6	
AST-120, CVD(−)	24.2	22.6	
non-AST-120, CVD(−)	11.2	8.4	0.0063
AST-120, CVD(+)	27.2	12.8	
non-AST-120, CVD(+)	9.8	2.9	<0.0001

**Table 5:** Uni- and multivariate analyses of the delaying effect of AST-120 on dialysis initiation. Uni- and multivariate analyses showed anemic status, eGFR, and not receiving AST-120 medication as independent factors associated with a significant increase in risk of dialysis initiation.

Univariate analysis	*P* value	Hazard ratio	95% CI
Age, <66.2, versus ≥66.2	0.808	0.979	0.828–1.158
CVD, without versus with	0.883	1.013	0.828–1.158
DM, without versus with	0.918	1.009	0.854–1.191
Systolic BP, <140, versus ≥140	0.228	1.123	0.930–1.355
BMI, <22, versus ≥22	0.960	1.004	0.846–1.193
ACEls/ARBs, without versus with	0.778	1.026	0.859–1.225
Ca blocker, without versus with	0.414	0.902	0.705–1.155
Activated Vit-D, without versus with	0.725	0.968	0.809–1.159
Hb, <8.8, versus ≥8.8	0.064	0.853	0.721–1.010
Alb, <3.6 versus, ≥3.6	0.996	1.000	0.846–1.183
Corrected Ca, <8.4 versus ≥8.4	0.681	1.036	0.875–1.227
Urine protein (mg/dL)	0.087	1.000	1.000–1.001
Urine protein (g/day)	0.488	1.001	0.999–1.003
eGFR, <8.0, versus ≥8.0	0.007	0.784	0.657−0.937
AST-120, with versus without	0.000	1.812	1.530–2.145

Multivariate analysis			

Hb, <8.8, versus ≥8.8	0.042	0.837	0.705–0.994
eGFR, <8.0, versus ≥8.0	0.005	0.773	0.645–0.925
AST-120, with versus without	0.000	1.881	1.586–2.231

**Table 6:** The 3-, 5-, and 10-year survival rate of pair-matched patients. The 3-, 5-, and 10-year survival rates from administration of AST-120 were analyzed by the Kaplan-Meier method with a log-rank test. Survival rates at 3-, 5-, and 10-years were not significantly different in the AST-120 and non-AST-120 groups.

Survival	3-year	5-year	10-year	*P* value
AST-120	79.0	63.8	29.8	0.0664
non-AST-120	78.1	65.6	44.0	

## Abbreviations

CKD: Chronic kidney disease
BMI: Body mass index
Hb: Hemoglobin
sCr: Serum creatinine
BUN: Blood urea nitrogen
eGFR: Estimated glomerular filtration rate
Alb: Albumin
IP: Inorganic phosphate
Ca: Calcium
IP: Inorganic phosphorus
BP: Blood pressure
CVD: Cardiovascular disease
DM: Diabetes mellitus
Vit D: Vitamin D
ACEIs: Angiotensin-converting enzyme inhibitors
ARBs: Angiotensin receptor blockers.
